# The Portuguese version of the body investment scale: psychometric properties and relationships with disordered eating and emotion dysregulation

**DOI:** 10.1186/s40337-020-00302-7

**Published:** 2020-07-01

**Authors:** Ana Isabel Vieira, Joana Fernandes, Paulo P. P. Machado, Sónia Gonçalves

**Affiliations:** Psychology Research Center (CIPsi), School of Psychology, University of Minho, 4710-057 Braga, Portugal

**Keywords:** Body image, Body investment, Eating disorders, Reliability, Psychometrics

## Abstract

**Background:**

This study aimed to examine the psychometric properties of the Portuguese version of the Body Investment Scale (BIS) in a nonclinical sample of students and a clinical sample of outpatients with eating disorders, to analyse the differences in the BIS factors between the samples and to explore the relationships among body investment, eating disorder symptoms and difficulties in emotion regulation.

**Methods:**

The clinical (*n* = 93) and nonclinical (*n* = 448) samples completed self-report measures.

**Results:**

In contrast to the nonclinical sample, confirmatory factor analysis showed an acceptable fit for the original four-factor solution of the BIS in the clinical sample. This scale also demonstrated adequate internal consistency in both samples. Significant differences in BIS factors were found between the samples; outpatients with eating disorders presented more negative feelings about the body, less comfort with touch and lower levels of body protection than those of the students. In the clinical sample, significant relationships were found between these factors and a higher severity of disordered eating, as well as between these factors and higher difficulties in emotion regulation.

**Conclusions:**

The Portuguese version of the BIS is a psychometrically sound measure for the assessment of body investment, and it is especially appropriate in a clinical setting of outpatients with eating disorders.

## Plain English summary

Few studies have assessed body investment (which includes feelings and attitudes about the body, comfort with being touched by other people, body care and protection) among individuals with eating disorders (EDs). Body dissatisfaction has received more research attention, and the relationship between body investment and EDs remains unclear. The Body Investment Scale (BIS) seems appropriate for evaluating body investment, but this scale has not been used in Portuguese research. We aimed to examine the psychometric properties of the Portuguese version of the BIS in a nonclinical sample of 448 students and a clinical sample of 93 outpatients with EDs, to evaluate the differences in the factors of the BIS between the samples and to explore the relationships among body investment, ED symptoms and difficulties in emotion regulation in the clinical sample. A confirmatory factor analysis supported the factor structure of the original BIS among Portuguese outpatients with EDs. Furthermore, this scale demonstrated adequate internal consistency, and outpatients with EDs presented more negative feelings and attitudes about the body, touch and lower body protection than those of the students. These factors were related to a high severity of disordered eating and more difficulties in emotion regulation. In conclusion, the BIS can be recommended for clinical use, especially in the ED context.

## Background

Body image, defined as a multifaceted experience of embodiment, including perceptions and attitudes towards one’s own body [1], has been the focus of research over time, especially regarding the self-evaluation of one’s body image. This experience is characterized by evaluative thoughts and beliefs about the appearance, involving self-ideal discrepancies and body satisfaction-dissatisfaction [2].

In particular, the relationship between body dissatisfaction and disordered eating has been studied in community and clinical settings. For example, a literature review by Lantz et al. [3] suggests that healthy women may overestimate their own body dimensions and have a propensity towards body dissatisfaction; the review also suggests that individuals with eating disorders (EDs) likely have a distorted body image, which in turn could contribute to a greater discrepancy between the actual and ideal self and facilitate the development and maintenance of ED symptomatology. Moreover, a self-evaluation influenced by body shape and weight is a central feature of anorexia and bulimia nervosa [4], and although the overvaluation of shape and weight is not necessary to diagnose binge eating disorder, patients with this problem who experience clinical overvaluation of shape and weight report greater levels of eating-related psychopathology than those with subclinical overvaluation [5]. Thus, a negative body image or dissatisfaction towards one’s appearance is considered a risk factor for the development of EDs [6, 7].

Despite the focus on body image evaluation, there has been a growing interest in the concept of body image investment, which concerns the cognitive and behavioural dimensions of one’s appearance, including an individual’s investment in beliefs or assumptions about the importance, meaning, and influence of appearance in life or for oneself, as well as behaviours related to the management or improvement of one’s appearance [2, 8].

Accordingly, existing measures assess several aspects of the body-image construct. For example, the Multidimensional Body-Self Relations Questionnaire (MBSRQ) [9] is a self-report inventory for the assessment of self-attitudinal aspects of body image, including evaluative, cognitive, and behavioural components. The MBSRQ reflects seven factors: Appearance Evaluation; Appearance Orientation; Fitness Evaluation; Fitness Orientation; Health Evaluation; Health Orientation; and Illness Orientation. This measure still has three subscales: Body Areas Satisfaction Scale, Overweight Preoccupation Scale, and Self-Classified Weight Scale [9, 10]. In the MBSRQ Users’ Manual, derived from the large normative samples, all Cronbach’s alphas are at satisfactory levels, and there is support for the convergent and discriminant validity of subscales in relation to other body image measures [9].

Another recognized instrument is the Appearance Schemas Inventory (ASI) [11] that assesses body image investment in relation to certain beliefs or assumptions about the importance, meaning and influence of appearance in one’s life. A revised version of this inventory (ASI-R) [8] included two factors: Self-Evaluative Salience and Motivational Salience. For both genders, the ASI-R and its two factors had high internal consistency and were convergent with other measures of body image and psychosocial functioning [8].

Nevertheless, these measures do not focus on the four aspects of the emotional investment in the body of the Orbach and Mikulincer’s Body Investment Scale (BIS) [12]: positive body image feelings and attitudes (e.g., “I am satisfied with my appearance”), comfort with being touched by other people (e.g., “I like to touch people who are close to me”), care with one’s body (e.g., “I believe that caring for my body will improve my well-being”) and protection of one’s body (e.g., “I look in both directions before crossing the street”).

Individuals with positive emotional investment in the body are more likely to engage in self-preservation behaviours than they are in self-destructive behaviours [12]. According to Orbach [13], a distorted body image and negative attitudes and feelings towards one’s body are related to anguish, hopelessness and stress and contribute to self-destructive behaviours, such as suicide and nonsuicidal self-injury (NSSI).

In a certain way, disordered eating behaviours can also be indirect forms of self-harm [14, 15] and a threat to the body. However, few studies have assessed body investment, as assessed by the BIS [12], in the context of EDs. Nevertheless, prior research showed that variables concerning body image investment (i.e., appearance orientation and importance) were associated with ED symptoms [16]. Moreover, individuals with anorexia nervosa, bulimia nervosa and body dysmorphic disorder reported more dysfunctional beliefs about the influence of physical appearance on one’s personal or social worth and sense of self, as well as a greater emphasis on being attractive and managing one’s appearance [17].

In the field of ED assessment, Portuguese versions of well-established measures, such as the Body Shape Questionnaire [18, 19], the Eating Disorder-15 questionnaire [20, 21] and the Eating Disorders Examination Questionnaire [22, 23], focus on body image evaluation. However, body-directed experiences, feelings and attitudes do not seem to be limited to body dissatisfaction, body weight and shape concerns in either ED or nonclinical samples. Therefore, self-report measures such as the BIS [12] seem more appropriate for evaluating investment in the body and for contributing to the assessment of body image as a multifaceted experience [1].

As far as we know, the BIS [12] has not been used in Portuguese research, and only four studies [12, 24–26] have examined the psychometric properties of this scale. In the original validation study [12], with a mixed sample of Israeli suicidal young people and community volunteers, an exploratory factor analysis revealed that the four factors explained 55% of the variance of body investment. The scale also demonstrated appropriate levels of internal consistency. Negative body attitudes and feelings, discomfort with touch, low care for the body, and reduced body protection distinguished between suicidal and non-suicidal adolescents, and positive body investment was related to lower suicidal tendencies. The BIS factors were significantly associated with physical anhedonia, perceived maternal care, overprotection, and self-esteem [12].

Two studies were conducted among American adolescents [26]: in the first study, with 204 high school adolescents and 197 psychiatric inpatient adolescents, a confirmatory factor analysis (CFA) showed a moderate fit for the four-factor solution of the BIS; in the second study, with 205 inpatient adolescents, improved fit estimates were found for the same solution. The BIS scales also revealed good-to-strong values of internal consistency, and body image feelings and body protection scores were useful in differentiating the responses of suicidal and non-suicidal adolescents. The concurrent validity was demonstrated by the significant correlations between the BIS scores and measures of reasons for living, attraction to death and attraction to life [26].

In the validation of the Brazilian version of the BIS, with a sample of 317 female students, the four-factor structure was confirmed [24]. However, the body protection factor revealed low internal consistency (*α =* .37) and was not correlated with the remaining three factors [24]. Finally, the Spanish version of the BIS was evaluated with 250 women diagnosed with an ED [25]. A CFA for the original BIS [12] showed a poor fit, but a respecified model showed an acceptable fit after correlating the measurement errors for items 3 (“It makes me feel good to do something dangerous”) and 7 (“I am not afraid to engage in dangerous activities”) and including item 12 (“I enjoy taking a bath”) into the body image feelings and attitudes factor. Estimates of internal consistency ranged from strong to acceptable, and the correlations between meaning of life and positive body image feelings, body touch, and body protection factors were significant and positive. On the other hand, the correlations between hopelessness and positive body image feelings, body protection, and body touch factors were significant and negative. The correlations between meaning of life and hopelessness and body care were not significant. Analysing the differences between groups with and without NSSI, patients who reported EDs and NSSI in the past year showed more negative body image feelings, higher discomfort with being touched by other people and lower body protection than patients without NSSI. Patients with and without suicidal ideation in the past year also differed on the same three BIS factors. Non-significant differences between groups were found regarding the body care factor [25].

Considering the lack of Portuguese measures to evaluate body investment, research is warranted to examine the fit of the factorial structure of the BIS and other psychometric properties of this measure in Portuguese samples. A measure capable of assessing the whole bodily experience can contribute to the identification of risk factors for self-harm behaviours, inform prevention and treatment practices, and facilitate an assessment of body image beyond its evaluation component.

Additionally, it will be important to compare the performance of the BIS factors between clinical and nonclinical samples and to analyse specific correlates of this measure. In this last domain, emotion regulation is expected to play an important role among individuals with EDs.

According to Gratz and Roemer’s multidimensional model [27], emotion regulation is characterized by four dimensions: (i) use of adaptive strategies to modulate emotional intensity or duration; (ii) maintaining behavioural control when distressed; (iii) emotional awareness, clarity and acceptance; and (iv) willingness to experience emotional distress to pursue meaningful activities. Anorexia and bulimia nervosa have been characterized by emotion regulation deficits [28]. In particular, EDs characterized by binge eating and purging are related to difficulties in maintaining behavioural control when distressed [29], and compared to a student group, patients with EDs report greater levels of emotion dysregulation, especially lower emotional awareness and clarity [30].

A prior study also suggested that individuals with EDs experience difficulties in identifying and managing their emotional experiences and that body dissatisfaction and one’s psychological investment in body image are predictive of disordered eating behaviours [31]. In addition, there is evidence that the relationship between body dissatisfaction and eating pathology is partially explained by negative affect [32]. It is possible that disordered eating and difficulties in emotion regulation may contribute to negative body-directed experiences, feelings and attitudes that are unrelated to body dissatisfaction.

Therefore, the present study aimed to (i) evaluate the fit of the four-factor structure of the BIS to a Portuguese nonclinical sample of students and a clinical sample of outpatients with ED; (ii) explore the internal consistency of the BIS factors in both samples; (iii) analyse the differences in the BIS factors between the nonclinical sample of students and the clinical sample of outpatients with EDs; and (iv) explore the relationships among body investment, ED symptoms and difficulties in emotion regulation in the clinical sample, including examining the extent to which ED symptoms and emotion regulation explain the variance of different facets of body investment. Consistent with previous literature, it was hypothesized that the Portuguese version of the BIS would have adequate psychometric properties. It was also hypothesized that the clinical sample would have more negative body image feelings and attitudes, higher discomfort with being touched by other people and lower body care and protection than the nonclinical sample. Finally, negative feelings and attitudes about the body, discomfort with being touched by other people, and low body care and protection would be related to a high severity of ED symptoms and more difficulties in emotion regulation.

## Methods

### Design

This is a cross-sectional study.

### Participants

A total of 541 participants completed this study. These participants were drawn from clinical (*n* = 93) and nonclinical (*n* = 448) samples.

The clinical sample – outpatients – consisted of 90 females and 3 males diagnosed with EDs, ranging from 14 to 31 years old (*M* = 22.42, *SD* = 4.01). Most participants were single (*n* = 88, 94.6%) and students (*n* = 52, 57.8%). Forty participants (43.5%) had a college-level education, 36 (39.1%) had a high school-level education, and 16 (17.4%) had a middle school-level education. Body mass index (BMI; kg/m^2^) ranged from 11.57 to 37.81 with a mean of 19.31 (*SD* = 4.73). According to the Diagnostic and Statistical Manual of Mental Disorders (fifth edition) criteria for EDs [4], 38 participants (41.8%) were diagnosed with anorexia nervosa restricting type, 6 (6.6%) were diagnosed with anorexia nervosa binge-eating/purging type, 27 (29.7%) were diagnosed with bulimia nervosa, 2 (2.2%) were diagnosed with binge ED, and 18 (19.8%) were diagnosed with other specified feeding or ED.

The nonclinical sample included 351 college students and 97 high school students. The mean age of the nonclinical sample was 19.61 years old (*SD* = 2.92; range = 14–26), and the majority were female (*n* = 353, 78.8%) and single (*n* = 443, 98.9%). The mean BMI (kg/m^2^) was 22.11 (*SD* = 3.32), and BMI ranged from 14.87 to 40.

### Measures

#### Sociodemographic questionnaire

This measure was developed by the research team to collect information about participants’ age, gender, marital status, level of education and occupation.

#### Body investment scale (BIS)

The BIS [12] is a self-report measure that assesses emotional investment in the body (i.e., body-directed experiences, feelings and attitudes) through 24 items, using a Likert-type scale ranging from 1 (I completely disagree) to 5 (I completely agree). It is divided into four factors with six items in each factor: feelings and attitudes towards body image (body image), comfort with being touched by other people (body touch), body care and body protection. Higher scores represent more positive feelings and attitudes about the body, greater comfort with being touched by other people, and higher levels of body care and protection. Orbach and Mikulincer [12] found Cronbach’s alpha values ranging from .75 to .92.

#### Eating disorder examination questionnaire (EDE-Q)

The EDE-Q [22, 23] is a self-report measure of 28 items that assesses the frequency and severity of ED symptoms over the past 28 days using a Likert-type scale ranging from 0 (no day) to 6 (every day). A global score is obtained from averaging the scores of four subscales: restraint, eating concern, weight concern and shape concern. The original version presented good psychometric properties [22]. The Portuguese version of the instrument showed good internal consistency, with Cronbach’s alpha values of .94 and 97 for the global score in community samples [23]. In the present study, Cronbach’s alpha for the EDE-Q global score (clinical sample *α* = .97; nonclinical sample *α* = .95) also indicate high internal consistency.

#### Difficulties in emotion regulation scale (DERS)

The DERS [27, 33] contains 36 items rated on a Likert-type scale ranging from 1 (almost never) to 5 (almost always) that assess emotion regulation difficulties across six domains: limited access to emotional regulation strategies (Strategies); non-acceptance of emotional responses (Non-acceptance); impulse control difficulties (Impulses); lack of emotional awareness (Awareness); difficulties engaging in goal-directed behaviour (Goals); and lack of emotional clarity (Clarity). Coutinho et al. [33] showed a high internal consistency for the total score (*α* = .93) in the Portuguese validation of the DERS. In the present study, Cronbach’s alpha for the total score was .96 in the clinical sample and .95 in the nonclinical sample.

### Procedure

This research was authorized and approved by the University of Minho Ethics Commission - Subcommittee of Ethics for Social and Human Sciences and the Portuguese Ministry of Education and was conducted in accordance with the Helsinki declaration.

The clinical sample was referred by clinicians from a public psychiatric service that provides specialized treatment for EDs. Participants were diagnosed by psychiatrists according to the DSM-5 criteria [4]. Regarding the nonclinical sample, college students were recruited from several Portuguese universities, mostly from the University of Minho, and high school students were recruited from a single school in northern Portugal across six classes from 9th to 12th grade. All participants were informed about the research aims, data confidentiality was assured, and participation was voluntary. Legal guardians and adults provided written informed consent.

The original English version of the BIS [12] was translated by the authors of this study. Then, a fluent bilingual (English and Portuguese) psychologist carried out a backtranslation. Both versions were compared, and discrepancies were analysed to clarify the Portuguese BIS. This version, which was used in the current study, is shown in Table 1.

**Table 1:** Original version and Portuguese translation of the BIS

Factor	Item
Body Image/Imagem Corporal	5. I am frustrated with my physical appearance (R)/Estou frustrado/a com a minha aparência física
	10. I am satisfied with my appearance/Estou satisfeito/a com a minha aparência
	13. I hate my body (R)/Odeio o meu corpo
	16. I feel comfortable with my body/Sinto-me confortável com o meu corpo
	17. I feel anger toward my body (R)/Tenho raiva do meu corpo
	21. I like my appearance’in spite of its imperfection/Gosto da minha aparência, apesar das imperfeições
Body Touch/Toque Corporal	2. I don’t like it when people touch me (R)/Não gosto quando as pessoas me tocam.
	6. I enjoy physical contact with other people/Gosto do contacto físico com as outras pessoas.
	9. I tend to keep a distance from the person with whom I am talking (R)/Costumo manter distância da pessoa com quem estou a falar
	11. I feel uncomfortable when people get too close to me physically (R)/Sinto-me desconfortável quando as pessoas se aproximam muito de mim fisicamente
	20. I like to touch people who are close to me/Gosto de tocar nas pessoas que estão próximas de mim
	23. Being hugged by a person close to me can comfort me/Pode conforta-me ser abraçado/a por uma pessoa que me é próxima
Body Protection/Proteção Corporal	7. I am not afraid to engage in dangerous activities (R)/Não tenho medo de participar em atividades perigosas
	3. It makes me feel good to do something dangerous (R)/Faz-me sentir bem fazer algo perigoso
	18. I look in both directions before crossing the street/Olho para ambos os lados antes de atravessar a rua
	7. I am not afraid to engage in dangerous activities (R)/Não tenho medo de participar em atividades perigosas
	15. When I am injured, I immediately take care of the wound/Quando me magoo, trato imediatamente da ferida
	22. Sometimes I purposely injure myself (R)/Às vezes, magoo-me de propósito
	24. I take care of myself whenever I feel a sign of illness/Cuido-me sempre que sinto um sinal de doença
Body Care/Cuidado Corporal	1. I believe that eating for my body will improve my well-being/Acredito que cuidar do meu corpo irá melhorar o meu bem-estar
	4. I pay attention to my appearance/Dou atenção à minha aparência
	8. I like to pamper my body/Gosto de mimar o meu corpo
	12. I enjoy taking a bath/Gosto de tomar banho
	14. In my opinion it is very important to take care of the body/Na minha opinião, é muito importante cuidar do corpo
	19. I use body care products regularly/Uso regularmente produtos de cuidado corporal

*Note.* R = reversed itens

### Statistical analyses

Descriptive statistics were obtained for samples’ sociodemographic characteristics. Separate confirmatory factor analyses (CFAs) were conducted on the nonclinical sample and the clinical sample to assess the goodness-of-fit of the original BIS [12] to the data using the maximum likelihood (ML) method for the model estimation. Participants who presented missing data were excluded from this analysis. Skewness (*Sk*) and kurtosis (*Ku*) were evaluated for all items of the BIS to assess the assumption of normality. According to Kline [34], absolute values of *Sk* > 3 and *Ku* > 10 indicate non-normal distributions. Based on these criteria, no non-normal distributions were found. We examined relevant fit estimates. Because the chi-square test (χ^2^/ degrees of freedom, *df*) is affected by the sample size and the size of the correlations in the model [35], alternative measures of fit were considered, such as the incremental fit index (IFI), the comparative fit index (CFI) and the root mean square error of approximation (RMSEA). IFI and CFI values >.90 were considered good, and RMSEA values < .07 were considered acceptable [34, 36]. To improve the overall model fit, modification indexes (MI) were examined. Covariances between items’ residuals suggested by higher MI values were allowed whenever there was a similar meaning of the items in each pair.

The internal consistency of the BIS factors was examined for the nonclinical and clinical samples by computing Cronbach’s alphas. To examine the discriminative efficiency of the BIS, independent sample *t* tests (*t*) were conducted to assess differences between the clinical sample of ED outpatients and the nonclinical sample of students regarding the BIS factors. To explore the relationships among body investment, ED symptoms and difficulties in emotion regulation in the clinical sample, we examined Pearson’s (*r*) and Spearman’s (*r*_*s*_) correlation coefficients and conducted multiple stepwise regressions. Each time a predictor is added to the model in the stepwise method, the least useful predictor is then removed. Thus, the regression is reassessed to find the best predictors and to verify whether any redundant predictors can be removed [37]. The relevant assumptions of this statistical analysis were tested.

CFA was conducted using IBM® SPSS® Amos™ 24.0, and other analyses were performed using IBM® SPSS® Statistics 24.0. *P* values < .05 were considered significant.

## Results

### Confirmatory factor analysis

A CFA for the BIS was performed on the nonclinical and clinical samples. In the nonclinical sample, the original four-factor solution showed a poor fit to the data, χ^2^/*df* = 1087.684, *p* < .001, IFI = .783, CFI = .781 and RMSEA = .087. To improve the model fit, MIs were examined, and the covariance between four pairs of items’ residuals was allowed: items 3 (“It makes me feel good to do something dangerous”) and 7 (“I am not afraid to engage in dangerous activities”); items 9 (“I tend to keep a distance from the person with whom I am talking”) and 11 (“I feel uncomfortable when people get too close to me physically”); items 3 (“It makes me feel good to do something dangerous”) and 22 (“Sometimes I purposely injure myself”); and items 22 (“Sometimes I purposely injure myself”) and 24 (“I take care of myself whenever I feel a sign of illness”). These correlations might be due to the similar meaning of the items in each pair. The respecified model also demonstrated a poor fit to the data, χ^2^/*df* = 860.565, *p* < .001, IFI = .841, CFI = .839 and RMSEA = .076.

In the clinical sample, initially, the original four-factor solution showed a poor fit to the data, χ^2^/*df* = 389.698, *p* < .001, IFI = .859, CFI = .855 and RMSEA = .083. To improve the model fit, MIs were examined, and the covariance between four pairs of items’ residuals was allowed: items 3 (“It makes me feel good to do something dangerous”) and 7 (“I am not afraid to engage in dangerous activities”, MI = 27.99); items 9 (“I tend to keep a distance from the person with whom I am talking”) and 11 (“I feel uncomfortable when people get too close to me physically”, MI = 6.64); items 3 (“It makes me feel good to do something dangerous”) and 22 (“Sometimes I purposely injure myself”, MI = 5.88); and items 1 (“I believe that eating for my body will improve my well-being”) and 8 (“I like to pamper my body”, MI = 5.17). These correlations might be due to the similar meaning of the items in each pair. The final respecified model showed an improved fit, χ^2^/*df* = 334.805, *p* < .001, IFI = .909, CFI = .906 and RMSEA = .068.

### Internal consistency

Cronbach’s alphas of the BIS factors were calculated in both the nonclinical and clinical samples. For the nonclinical sample, internal consistency was excellent for the body image factor (*α* = .91), good for the body touch factor (*α* = .80), and acceptable for both the body protection (*α* = .66) and body care factors (*α* = .62).

For the clinical sample, internal consistency was excellent for the body image factor (*α* = .93), good for both the body touch (*α* = .83) and body protection factors (*α* = .76), and acceptable for the body care factor (*α* = .67).

### Differences between samples in the BIS factors

Table 2 presents the mean scores on the BIS factors for the clinical and nonclinical samples. In comparison with the nonclinical sample, participants with EDs reported more negative feelings and attitudes about the body, *t* (536) = 10.77, *p* < .001, higher discomfort with being touched by other people, *t* (534) = 6.53, *p* < .001, and lower degree of body protection, *t* (537) = 6.03, *p* < .001. Non-significant differences between the samples were found regarding the body care factor, *t* (538) = 1.58, *p* = .115.

**Table 2:** Mean scores on the BIS factors for the clinical and nonclinical samples

	Clinical (*n* = 93)	Nonclinical (*n* = 448)	*t*
	*M (SD)*	*M (SD)*	
BIS			
Body Image	2.61 (1.02)	3.78 (.92)	10.77^***^
Body Touch	3.13 (.77)	3.71 (.76)	6.53^***^
Body Protection	3.61 (.82)	4.05 (.59)	6.03^***^
Body Care	4.10 (.55)	4.20 (.52)	1.58

^***^p < .001

### Relationships among body investment, ED symptoms and difficulties in emotion regulation

As shown in Table 3, among the participants with EDs, the correlations between the EDE-Q subscales and body image, body touch, and body protection factors were significant and negative. Negative attitudes and feelings towards the body, less comfort with physical contact and low levels of body protection were correlated with a high severity of disordered eating. No significant correlations were found between the body care factor and the EDE-Q subscales.

**Table 3:** Correlations among the body investment, ED symptoms and difficulties in emotion regulation in the clinical sample

	EDE-Q Restraint^a^	EDE-Q Eating concern^a^	EDE-Q Weight concern^a^	EDE-Q Shape concern^a^	EDE-Q Global Score^a^	DERS Strategies	DERS Non-acceptance^a^	DERS Awareness	DERS Impulses	DERS Goals^a^	DERS Clarity	DERS Total Score
BIS												
Body Image	−.60^***^	−.53^***^	−.68^***^	−.74^***^	−.73^***^	−.52^***^	−.33^**^	−.27^**^	−.43^***^	−.46^***^	−.31^**^	−.51^***^
Body Touch	−.52^***^	−.43^***^	−.45^***^	−.51^***^	−.54^***^	−.40^***^	−.44^***^	−.27^*^	−.31^**^	−.34^***^	−.30^**^	−.44^***^
Body Protection	−.49^***^	−.41^***^	−.51^***^	−.51^***^	−.57^***^	−.31^**^	−.23^*^	−.26^*^	−.27^*^	−.30^**^	−.28^**^	−.36^***^
Body Care	−.15	−.09	−.18	−.21^†^	−.21^†^	−.32^**^	−.27^*^	−.31^**^	−.25^*^	−.32^**^	−24^*^	−.35^***^

Note: ^a^Spearman’s correlation coefficients. The remaining values represent Pearson’s correlation coefficients. ^†^*p* < .10; *p < .05; **p < .01; ***p < .001

Regarding the correlations between body investment and difficulties in emotion regulation, significant and negative correlations were found between the BIS factors and the DERS subscales. Lower scores on all factors of the BIS were correlated with more difficulties in all dimensions of emotion regulation: limited access to emotional regulation strategies, non-acceptance of emotional responses, lack of emotional awareness, impulse control difficulties, difficulties engaging in goal-directed behaviour, and lack of emotional clarity.

In the clinical sample, four multiple stepwise regression analyses were conducted using each of the BIS factors as dependent variables and age, BMI, the EDE-Q global score and the DERS total score as independent variables. The predictor variables were entered in the following order: age and BMI (Step 1), the EDE-Q global score (Step 2), and the DERS total score (Step 3). In each regression, age and BMI did not significantly contribute to the variance of the outcome variables. For this reason, these variables were removed from the final models, and the models were re-estimated for the remaining predictors. The final multiple stepwise regression models were significant, explaining between 22 and 50% of the variance of body image, body touch, body protection and body care (Table 4). Specifically, only the EDE-Q global score was associated with body image, body touch and body protection factors, whereas the DERS total score was the only variable that was associated with the body care factor. A higher severity of disordered eating behaviours was related to more negative body image feelings and attitudes, less comfort with touch and lower levels of body protection. On the other hand, a higher level of emotion dysregulation was related to lower body care.

**Table 4:** Final models of multiple stepwise regressions for the BIS factors in the clinical sample

	Body Image^b^				Body Touch^c^				Body Protection^d^				Body Care^e^			
	*R*^*2*^	*B*	*SE B*	*β*	*R*^*2*^	*B*	*SE B*	*β*	*R*^*2*^	*B*	*SE B*	*β*	*R*^*2*^	*B*	*SE B*	*β*
Model	.50				.28				.35				.22			
Constant		3.79	.16			3.81	.15			4.38	.14			4.97	.20	
EDE-Q Global Score		−.43	.05	−.71^***^		−.24	.05	−.53^***^		−.28	.04	−.59^***^		–	–	–
DERS Total Score		–	–	–		–	–	–		–	–	–		−.01	.00	−.46^***^

*Note: Note:*^b^*ΔR*^2^ = .50 (*p* < .001). ^c^*ΔR*^2^ = .27 (*p* < .001). ^d^*ΔR*^2^ = .34 (*p* < .001). ^e^*ΔR*^2^ = .20 (*p* < .001). The unfilled table cells (“-”) represent predictors that do not make a statistically significant contribution to how well the model predicts the outcome variable. In such cases, following the stepwise method, the least useful predictors are removed from the model and the model is re-estimated for the remaining predictors. For this reason, age and BMI were also removed from all models. ***p < .001

## Discussion

The main aim of this study was to examine the psychometric properties of the Portuguese version of the BIS [12] in a nonclinical sample of students and a clinical sample of ED outpatients. The current study also aimed to analyse the differences between the nonclinical sample of students and the clinical sample of ED outpatients regarding BIS factors and to explore the relationships among body investment, ED symptoms and difficulties in emotion regulation.

The results of the CFA showed that the four-factor solution of the BIS, similar to the one obtained by Orbach and Mikulincer [12], showed a poor fit to the nonclinical sample of students. On the other hand, the four-factor solution of the BIS showed a moderate fit to the clinical sample of outpatients with EDs. Specifically, in the clinical sample, the initial findings revealed a poor fit to the data. However, a respecified model, including covariances between four pairs of item residuals due to the similar meaning of the items in each pair, showed an improved fit. Our results are consistent with the validation study of the Spanish version of the BIS among patients with EDs [25], in which measurement errors for items 3 (“It makes me feel good to do something dangerous”) and 7 (“I am not afraid to engage in dangerous activities”) were correlated to improve the goodness of fit. In contrast to the nonclinical sample, the CFA results also suggest that the four-factor solution of the BIS adequately describes aspects of the emotional investment in the body among individuals with EDs. Given that a negative body investment can be indicative of self-destructive behaviours [12], it is likely that the dimensions of the BIS may be especially represented among outpatients with EDs, who have a difficult relationship with their bodies, potentially leading to self-harm through behaviours such as eating restraint, excessive exercise and purging.

Regarding estimates of internal consistency, our findings agree with those reported in previous studies [25, 26]. For both samples, the highest Cronbach’s alpha value was observed for the body image factor, followed by the body touch and body protection factors. Although it was acceptable, the body care factor yielded the lowest Cronbach’s alpha value. This result can be explained by the fact that outpatients with EDs overvalue and take care of their bodies. Thus, the body care factor may be less representative in the clinical sample and in the nonclinical sample, since individuals from the community should also perform common body care behaviours such as taking a bath and using body care products. Additionally, items related to body care (e.g., “I believe that eating for my body will improve my well-being”; “In my opinion, it is very important to take care of the body”) might not represent a cohesive construct, especially in a sample of outpatients with EDs. Thus, similar to the study by Marco et al. [25], these reasons may explain why body care was not related to other variables and was not different between samples.

Given that EDs underlie negative attitudes towards one’s own body [38], ED outpatients reported more negative feelings about the body, higher discomfort with being touched by other people and lower body protection than participants from the nonclinical sample. Thus, body image, body touch and body protection factors were useful in differentiating the responses of ED outpatients and college and high school students. Our correlation analyses in the clinical sample also revealed that more negative attitudes and feelings towards the body, less comfort with physical contact and low levels of body protection were associated with a high severity of disordered eating. Therefore, difficulties in these facets of body investment may indicate more severe disordered eating behaviours.

On the other hand, previous evidence suggests that low investment in the appearance contributes to self-harm behaviours to cope with unpleasant emotions [39], and in the present study, a lower degree of body investment was correlated with more difficulties in all dimensions of emotion regulation.

In addition to the correlations, hierarchical multiple regression analyses showed that a higher severity of disordered eating behaviour was a potential predictor of negative body image feelings and attitudes, less comfort with touch and lower levels of body protection. On the other hand, more difficulties in emotion regulation were related to lower body care. Thus, this study suggests the relevance of assessing disordered eating severity and emotion dysregulation as potential risk factors for and/or correlates of lower investment in the body. Nevertheless, these results should be interpreted with caution given the cross-sectional nature of the current study.

Given the lack of measures specific to evaluating body investment in Portugal, the current study extended previous findings [12, 25] and was the first to examine the psychometric properties of the Portuguese version of the BIS using a nonclinical sample of students and a clinical sample composed of outpatients with anorexia nervosa, bulimia nervosa, binge eating disorder and other specified feeding or EDs. The results demonstrated that the Portuguese BIS is a psychometrically sound measure for the assessment of body investment. In addition, most BIS factors differed between the nonclinical sample and the clinical sample, and body investment seems to be related to more severe ED symptoms and difficulties in emotion regulation among ED outpatients.

Despite the relevance of this study, it is important to highlight some limitations. First, the small size of the clinical sample limits our knowledge about the structure of the BIS with outpatients with EDs. Nevertheless, our model was specified in a theoretically meaningful way, based on the original version of the BIS [12], and given that the chi-square test (χ^2^/degrees of freedom, *df*) is affected by the sample size and the size of the correlations in the model [35], alternative fit indexes were considered. Second, this study did not examine the convergent validity of the BIS with other validated body image scales or the test-retest reliability. Third, the samples were collected in northern Portugal, which inhibits the generalization of results to the entire Portuguese population. Additionally, both nonclinical and clinical samples contain different percentages of male respondents. Indeed, the BIS may operate differently in males and females, and the percentages obtained may contribute to the bias in the data collection. However, we believe that these percentages correspond to a true gender distribution in the sampled populations, namely, among outpatients with EDs, in which the number of males is quite small compared to that of females. Fourth, participants with EDs were undergoing outpatient treatment and represent only part of the general population with EDs. Finally, there is a reliance on self-report methods, and the cross-sectional design cannot address the causal relationships among body investment, ED symptoms and difficulties in emotion regulation. Our findings should be replicated, especially given the small size of the clinical sample. Future longitudinal studies are needed to examine the test-retest reliability of the Portuguese BIS and to confirm the relationships between the variables. Future studies should also explore the convergent validity of the BIS with other body image measures and consider using other samples with equal proportions of men and women.

## Conclusions

The current study suggests that the Portuguese version of the BIS [12] has adequate psychometric properties. Body investment may also be considered alongside other psychosocial variables to better understand behaviours that damage physical health. The BIS can extend and complement the existing Portuguese measures focused not only on body image evaluation but also on other important dimensions of the body experience, such as comfort with being touched by other people or body care. With a comprehensive assessment, researchers and clinicians expand conceptualizations and clinical formulations, and they can identify body disturbances, which in turn inform coping strategies for dealing with negative body image feelings and attitudes, discomfort with touch, low levels of body care and low levels of body protection. Consequently, efforts to improve body awareness [40, 41] and acceptance [42], as well as efforts to increase the ability to tolerate emotional discomfort and deal with unpleasant emotions, can be helpful in individuals who show negative emotional investment in the body. In sum, this study supports the usefulness of this scale to assess body investment, especially in the context of EDs.

## Data Availability

The datasets used and/or analysed during the current study are available from the corresponding author on reasonable request.

## Abbreviations

ED: Eating disorders
MBSRQ: Multidimensional Body-Self Relations Questionnaire
ASI: Appearance Schemas Inventory
ASI-R: Revised version the Appearance Schemas Inventory
BIS: Body Investment Scale
APA: American Psychiatric Association
CFA: Confirmatory factor analysis
BMI: Body mass index
EDE-Q: Eating disorder examination questionnaire
DERS: Difficulties in emotion regulation scale
DSM-5: Diagnostic and Statistical Manual of Mental Disorders (fifth edition)
ML: Maximum likelihood
IFI: Incremental fit index
CFI: Comparative fit index
RMSEA: Root mean square error of approximation
MI: Modification index
SPSS: The statistical package for the social sciences
